# The Level of Agreement between Self-Assessments and Examiner Assessments of Melanocytic Nevus Counts: Findings from an Evaluation of 4548 Double Assessments

**DOI:** 10.3390/curroncol31040164

**Published:** 2024-04-13

**Authors:** Olaf Gefeller, Isabelle Kaiser, Emily M. Brockmann, Wolfgang Uter, Annette B. Pfahlberg

**Affiliations:** Department of Medical Informatics, Biometry and Epidemiology, Friedrich-Alexander-Universität Erlangen-Nürnberg, 91054 Erlangen, Germany; isabelle.kaiser@fau.de (I.K.); wolfgang.uter@fau.de (W.U.); annette.pfahlberg@fau.de (A.B.P.)

**Keywords:** cutaneous melanoma, screening, melanocytic nevus, self-counting, eye color

## Abstract

Cutaneous melanoma (CM) is a candidate for screening programs because its prognosis is excellent when diagnosed at an early disease stage. Targeted screening of those at high risk for developing CM, a cost-effective alternative to population-wide screening, requires valid procedures to identify the high-risk group. Self-assessment of the number of nevi has been suggested as a component of such procedures, but its validity has not yet been established. We analyzed the level of agreement between self-assessments and examiner assessments of the number of melanocytic nevi in the area between the wrist and the shoulder of both arms based on 4548 study subjects in whom mutually blinded double counting of nevi was performed. Nevus counting followed the IARC protocol. Study subjects received written instructions, photographs, a mirror, and a “nevometer” to support self-assessment of nevi larger than 2 mm. Nevus counts were categorized based on the quintiles of the distribution into five levels, defining a nevus score. Cohen’s weighted kappa coefficient (κ) was estimated to measure the level of agreement. In the total sample, the agreement between self-assessments and examiner assessments was moderate (weighted κ = 0.596). Self-assessed nevus counts were higher than those determined by trained examiners (mean difference: 3.33 nevi). The level of agreement was independent of sociodemographic and cutaneous factors; however, participants’ eye color had a significant impact on the level of agreement. Our findings show that even with comprehensive guidance, only a moderate level of agreement between self-assessed and examiner-assessed nevus counts can be achieved. Self-assessed nevus information does not appear to be reliable enough to be used in individual risk assessment to target screening activities.

## 1. Introduction

Incidence rates of cutaneous melanoma (CM) have shown a dynamic development in countries with predominantly fair-skinned populations in recent decades [1]. In Scandinavian countries and New Zealand, where population-based cancer registries have documented trends over a long period since the 1940s, incidence rates have increased 30- to 40-fold over the last 70 years, with annual increases of 3–7% [1,2,3]. CM, which used to be a rare form of skin cancer until the middle of the last century, is currently an important public health issue in many countries worldwide.

CM is an obvious candidate for screening programs because its prognosis is excellent when it is diagnosed at an early stage of the disease [4,5,6]. Current evidence on the benefits of CM screening, summarized for the US Preventive Services Task Force in a recent systematic review covering 20 studies [7], is, however, inconsistent. Due to the high financial costs of population-wide screening for CM and the potential for overdiagnosis, i.e., the identification of otherwise indolent melanoma or false-positive melanoma diagnoses that turn out to be benign lesions [8], most countries have not issued a recommendation regarding CM screening thus far [9]. Only two countries have established national guidance on CM screening by physicians. Germany recommends visual skin examinations by dermatologists or general practitioners who have been certified for performing skin cancer screening. Examinations can take place every two years (beginning at age 35) and are covered by statutory health insurance [10]. France recommends that general practitioners assess the CM risk of their patients via a checklist consisting of seven questions and refer high-risk patients for further diagnostic clarification to a dermatologist [11].

An alternative to population-wide screening is targeted screening of those at high risk of developing CM [12]. An efficient implementation of such a strategy requires that the CM risk can be assessed simply, reliably, and validly. To this end, the number of melanocytic nevi has been consistently identified as a strong predictor of CM [13,14]. Nearly all prediction models developed to date to capture CM risk have incorporated the number of nevi, mostly in a categorized form, among the explanatory variables [15]. A prerequisite for using the number of nevi in order to identify individuals at high CM risk for targeted screening, however, is that this variable can be validly assessed. The implementation of targeted screening is particularly cost-efficient if self-assessment of nevi is used. Otherwise, physicians would need to be involved in identifying the high-risk group for actual screening, and potential savings compared to population-wide screening are reduced.

By addressing the aspect of validity when using self-assessment to identify the number of melanocytic nevi, the objective of our study was to analyze the level of agreement between self-assessment and examiner assessment of nevus counts based on a large dataset of study subjects in whom mutually blinded double counting of nevi was performed. We report on the quantitative degree of agreement between the two assessments and on potential determinants of the agreement.

## 2. Materials and Methods

### 2.1. Design and Participants

The study started in April 2006 as a repeated cross-sectional survey and was conducted in an identical way twice a year (in April and November) until November 2019 at the University of Erlangen–Nuremberg. The study was embedded as a practical exercise in a university course that introduced students to epidemiological and biostatistical methods. Students could only participate once in the study. Each semester, all students enrolled in the course “Biometry and Epidemiology” were invited to participate in the study. Of the 5125 students enrolled during the 13.5-year study period, 4768 students attended the course for the first time on the day when the practical exercise took place. Of these, 4704 (98.7%) agreed to participate and provided written informed consent. The study was approved by the Ethics Committee of the University of Erlangen–Nuremberg.

### 2.2. Nevus Counting Procedure

The criteria for the nevus counting procedure followed the IARC protocol for identifying and recording nevi [16]. Accordingly, all acquired melanocytic nevi with a diameter of at least 2 mm were eligible for counting. The assessment of nevi was performed by visual inspection of the skin without the assistance of epiluminescence microscopy. For feasibility reasons, nevus counting was limited to the area between the wrist and the shoulder, as nevus counts on the arms are highly correlated with total body counts [17]. The number of melanocytic nevi on both arms was counted twice, once by the participant and once by one of the six examiners involved in the study, in a mutually blinded manner; participants were not aware of the counting result of the examiner when performing the nevus counting and vice versa (for logistical reasons, the sequence of nevus counting was not identical for all participants; in one subgroup, examiners counted first, and in the other subgroup, examiners counted last).

### 2.3. Instructions for Nevus Self-Counting

Participants received written instructions prior to nevus counting. Additionally, they were given a mirror to facilitate counting on the dorsal upper areas of the arms and a “nevometer” [18], a small, 2 mm thick transparent polymethylmethacrylate spatula with a hole of 2 mm in diameter, to be used as a reference when deciding whether the size of a lesion was above the 2 mm threshold. The written instructions explained the nevus counting procedures in detail. On three pages supplemented by color photographs, the area where nevi should be counted was defined, the handling of the nevometer and mirror were explained, and the differentiation of melanocytic nevi from other skin lesions was described. The latter explanation, crucial for the validity of self-assessment, was supported by photographs of various skin lesions to clarify the appearance of melanocytic nevi and to distinguish them from other skin lesions, such as café au lait spots, Becker’s nevus, viral warts, freckles, hemangiomas, dermatofibromas, and other potentially similar lesions.

### 2.4. Standardization of Nevus Counting by the Examiners

The group of examiners consisted of six people altogether: one dermatologist, two physicians of other disciplines, and three academic researchers. The examiners involved in this study had extensive prior experience in performing assessments of melanocytic nevi due to their involvement in another epidemiological study [19], for which the non-dermatologists had received specific training from dermatologists. Standardization of the nevus counting procedure between all examiners who performed the assessments was essential for the study. To achieve this goal, regular training sessions were held before the study dates in each winter and summer semester, during which the examiners discussed all aspects of the nevus counting procedure and carried out practical exercises.

### 2.5. Questionnaire

In addition to nevus counting, information from the study participants was collected using a self-administered questionnaire that comprised sociodemographic variables (age, sex, and degree course) and phenotype information (hair and eye color, freckling, and skin type). Participants were provided with an eye and hair color reference chart, an illustration of different categories of freckling, and a detailed description of how to assess the Fitzpatrick skin type [20] in order to standardize the assessment of phenotype.

### 2.6. Statistical Analysis

Agreement between the two nevus assessments was analyzed based on (i) the raw results of the nevus counting and (ii) a categorized nevus score (categories defined by the quintiles of the nevus distribution). For these analyses, all participants with Fitzpatrick skin types V and VI were excluded. For the raw nevus counts, the mean difference (±standard deviation (SD)) between the two assessments and the intraclass correlation coefficient (ICC), accompanied by its 95% confidence interval (CI), were computed to quantify the relationship between the two nevus assessments. The ICC was estimated from generalized linear mixed models assuming a zero-inflated negative binomial distribution to take account of the overdispersed nevus count data [21]. Additionally, the differences between the two nevus counting results were examined graphically using a Bland–Altman plot, which displayed the difference in relation to the average count as a surrogate for the unknown true number of nevi. For the categorized nevus score, observed agreement (in percent) and Cohen’s weighted kappa coefficient (κ) using linear weights for the categories to incorporate the ordinal structure of the nevus score were estimated from the 5 × 5 contingency table showing the joint distribution of participants’ and examiners’ nevus classification [22,23]. The precision of these estimates is depicted by 95% CIs. Symmetry in this 5 × 5 contingency table was statistically tested using Bowker’s test [24].

To evaluate potential determinants of agreement, subgroups of the total study group were defined by the variables sex (male/female), degree course (clinical medicine/other), semester (summer/winter), skin type (Fitzpatrick type I–IV), freckles (yes/no), hair color (red, blonde, brown, or black), and eye color (dark blue, light blue, green, green-brown, light brown, or dark brown). Weighted kappa coefficients were estimated in the subgroups, and differences between subgroup-specific estimates were statistically evaluated for each of the seven variables by the test for equal weighted kappa coefficients between independent groups developed by Fleiss et al. [25]. *p*-values less than 0.05 were considered to indicate statistical significance but should be interpreted in an explorative manner as no adjustment for multiple testing was made. All statistical analyses were carried out using R software version 4.3.2 (R Foundation for Statistical Computing, Vienna, Austria). In particular, the R package irrCAC was used for estimating weighted kappa coefficients and the R package iccCounts for estimating the ICC.

## 3. Results

Of the recruited study group (*n* = 4704), 90 participants were excluded from the analysis due to their skin type (i.e., Fitzpatrick type V–VI), 48 had incomplete information about their nevus status because one of the assessments was missing, and data from an additional 18 participants with double counting results were excluded from the analysis because the examiner assessment was carried out by a one-time substitute examiner. The remaining 4548 participants comprised 1689 (37%) men, 2845 (63%) women, and 14 (0.3%) participants who did not reveal their gender. The participants’ mean (±SD) age was 23.53 (±3.36) years. Altogether, 4156 (91%) of the participants studied clinical medicine, and 392 (9%) students came from other disciplines (molecular medicine, life science engineering, and speech therapy). Nearly equal numbers of participants completed the study during the summer term (*n* = 2241, 49%) and the winter term (*n* = 2307, 51%). A descriptive summary of the distribution of phenotype variables in the study sample is given in Table 1.

### 3.1. Distribution of Nevus Counts

Both distributions, i.e., that of the self-assessed number of nevi and that of the examiner-assessed number of nevi, were strongly skewed. While the median numbers of self-assessed and examiner-assessed nevi were 14 and 12, respectively, the arithmetic means were 18.9 and 15.6, respectively. When defining the nevus score, i.e., the categorized version of the counted number of nevi, we used the joint distribution of all nevus counts from participants and examiners. The quintiles of this joint distribution were 5, 10, 16, and 26 nevi, leading to the following five categories for the nevus score: ≤5, (5–10], (10–16], (16–26], and >26.

### 3.2. Nevus Counts: Differences between Assessments

**Figure 1:**
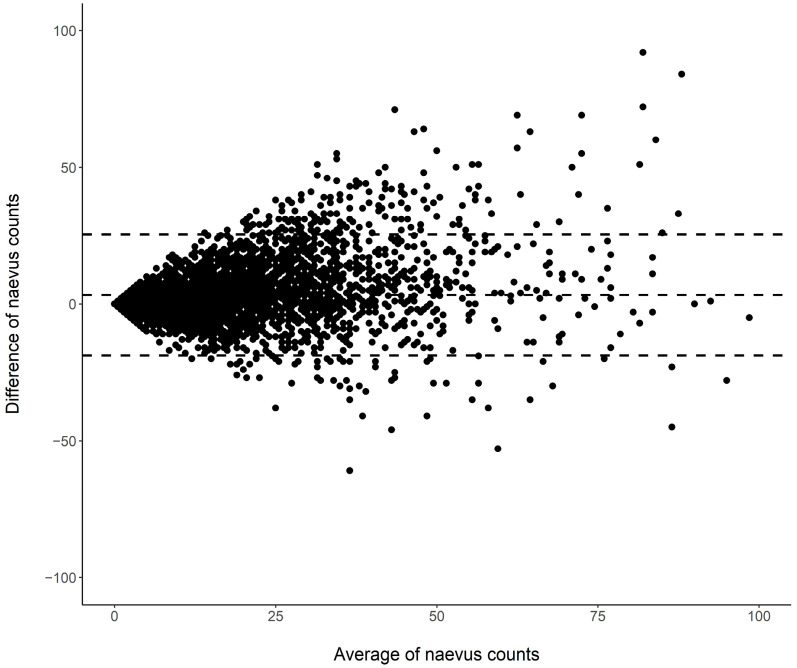
Bland–Altman plot showing the relationship between difference and average of the number of nevi counted by study participants and examiners in a mutually blinded fashion (*n* = 4539; nine data points with an average nevus count > 100 were outside the display range).

The results of self-assessed and examiner-assessed nevus counting showed an intraclass correlation of 0.810 (95% CI: 0.799–0.822). The self-assessed nevus counts were somewhat greater than the examiner-assessed counts, and the mean difference (±SD) in the total sample was 3.33 (±11.29). Figure 1 displays the Bland–Altman plot for the data, showing the individual mean differences in relation to the averages of the two counts.

### 3.3. Nevus Score: Agreement between Assessments

The joint distribution of the self-assessed and examiner-assessed nevus scores is shown in Table 2. Based on the five-level score, the observed agreement in the total sample was 50.29% (95% CI: 48.83–51.74%). The agreement as measured by the weighted kappa estimate was moderate (κ = 0.596, 95% CI: 0.581–0.611). Sensitivity analyses using a categorized nevus score with a different number of categories between 3 and 7 (using a data-adaptive definition of the specific categories, i.e., tertiles, quartiles, etc.) yielded stable results regarding estimated weighted kappa coefficients.

The disagreement between self-assessed and examiner-assessed nevus score categories was not symmetric (see Table 2). While 33.64% (95% CI: 32.27–35.01%) of the students chose a higher nevus score category than the examiners, in only 16.07% (95% CI: 15.01–17.14%) of the cases, the examiners chose the higher nevus category. The deviation from symmetry in the contingency table was statistically significant (*p* < 0.001).

The detailed results of the agreement between the two assessments in the subgroups defined by sociodemographic and phenotype variables are shown in Table 3. Overall, the level of agreement was quite homogenous across the subgroups. For all variables except eye color, we found a nonsignificant variation of limited magnitude in the weighted kappa estimates of the corresponding subgroups. However, in the subgroups defined by participants’ eye color, we observed a much lower weighted kappa of 0.512 (95% CI: 0.462–0.561) for those with dark blue eyes and a higher weighted kappa of 0.642 (95% CI: 0.598–0.685) for those with dark brown eyes, whereas the other four subgroups showed weighted kappas at approximately the same level as in the total sample. Heterogeneity in the level of agreement between the six subgroups defined by participants’ eye color reached significance (*p* = 0.01).

## 4. Discussion

In a standardized study setting, we evaluated nevus self-counting by comparing self-assessed and examiner-assessed nevus counts obtained in a mutually blinded fashion. Based on 4548 double counting results, we observed only a moderate level of agreement between the two assessments. Self-assessed nevus counts were slightly higher than those determined by trained examiners. The level of agreement was independent of sociodemographic and cutaneous factors. We, however, found a greater than average level of agreement in participants with dark brown eyes and a lower than average level of agreement in those with dark blue eyes. Participants’ eye color was the only factor that had a significant impact on the level of agreement in our study.

Tackling aspects of the validity and reliability of nevus self-counting is not a new topic in dermato-epidemiologic research. In a systematic literature search, we identified 21 studies published between 1991 and 2021 [18,26,27,28,29,30,31,32,33,34,35,36,37,38,39,40,41,42,43,44,45] that reported some data on the relationship between self-counting and examiner counting of nevi on the same individuals; 13 of these studies focused primarily on this topic. Comparisons of results between validation studies are complicated by strong differences in study design, nevus counting protocols, nevus definitions, statistical methods used to analyze the agreement, the sex and age distributions of participants, and whether counts were conducted on the whole body or on specific anatomical sites. All previous investigations were based on much smaller study samples, comprising between 46 [27] and 1772 [40] participants, which limits, at least in half of the previous studies with less than 250 participants, the statistical precision of quantifying the level of agreement. Most of them were embedded in studies with melanoma patients or used participants in screening programs for skin cancer as their study subjects. Unsurprisingly, the results of these studies were highly inconsistent because the studies differed in so many ways, most notably in the instructions given to study participants for nevus self-counting. The lowest level of agreement was observed in a study of 1658 employees of banks and insurance companies who had only a few minutes to fill out a self-administered questionnaire prior to a voluntary skin check by a dermatologist [43]. The questionnaire comprised one question asking participants to estimate the total number of moles on their body (0–10, 11–30, 31–50, 51–100, >100) without further explanation and instructions. These self-assessments of nevi showed virtually no agreement beyond chance with the results of nevus counting by the dermatologist (weighted κ = 0.03). Other studies have reported higher levels of agreement, which is more consistent with our findings. For example, Jackson et al.’s study [30] in the United Kingdom, comprising eight general practitioner practices with 388 participants, found an identical level of agreement (κ = 0.60) as our study for a three-level nevus score. A study by Mannino et al. [45] in three European dermatologic clinics that assisted participants in assessing their nevi with instructions and photographs similar to ours reported a moderate level of agreement (weighted κ = 0.45), slightly below our result, in their sample of 744 patients.

There have been conflicting findings regarding the direction of differences between self-assessed and examiner-assessed nevus counts. While two Australian validation studies [31,41] found that self-assessment of nevi underestimates the number of melanocytic nevi, the study by Lawson et al. [28] in the United States and the European study by Richtig et al. [35] observed an overestimation of nevi when self-assessed. Our results corroborate the latter findings, i.e., self-assessed nevus counts were higher than examiner-assessed counts. Students evaluated more skin lesions as being melanocytic nevi larger than 2 mm in diameter than the examiners. Our data do not allow us to draw clear conclusions as to the reasons for this overcounting and which types of skin lesions were mistakenly considered to be melanocytic nevi by the students (probably freckles, flat warts, and smaller café-au-lait spots) and which types of melanocytic nevi (e.g., Spitz nevi) may have been overlooked. However, based on the examiners’ impressions during the conduct of the nevus counting, we believe that the main reason for the overcounting of nevi by the students was that not all participants used the “nevometer” properly and counted nevi < 2 mm, which should not have been counted.

The vast majority (91%) in our study sample were students of clinical medicine attending a compulsory course in their degree program, while the remaining students attended the course as part of another degree program. Of course, such a sample is not representative of the general population. At the time of the study, however, the students had no previous dermatologic training and thus had no experience in distinguishing melanocytic nevi from other skin lesions. Nevertheless, we cannot exclude the possibility that their general medical training and attitudes toward this topic may have influenced their motivation and diligence when participating in the study, which may have biased the results toward a higher level of agreement in our study than would be expected in the general population. A further limitation relates to the fact that the examiner assessments of nevi were not performed by a single trained dermatologist assisted by epiluminescence microscopy but by a group of examiners that comprised only one dermatologist and five further physicians and academic researchers using only visual inspection of the skin without support of epiluminescence microscopy. The non-dermatological examiners involved in the study were specifically trained to perform nevus counting and had prior experience in this area from another study. Regular meetings of the group of examiners during the study period were held to ensure a high level of standardization in performing nevus counting by all examiners.

When self-assessed information on melanoma risk factors is used to identify a subgroup in the population at high risk of developing melanoma as a target of screening activities, the self-assessed information needs to be accurate. Although prediction models developed for estimating melanoma risk usually include several variables, the frequency of nevi is a particularly important component of such models [15]. From the literature on melanoma prediction models, there is some empirical evidence that the discriminatory properties of melanoma prediction models incorporating self-assessed nevus information are inferior to those using physician-assessed nevus information. Four publications [46,47,48,49] described melanoma prediction models derived from the same data, namely, the Australian Melanoma Family Study [50], and validated them using data from, again, the same population-based case-control study in the United Kingdom [51]. The prediction models differed with respect to the incorporated variables: two [48,50] used genotype information in addition to phenotype and UV exposure variables, while the remaining two [47,49] focused on nongenetic risk factors. The difference between the latter two models is related to the incorporation of only self-assessed phenotype variables in [47] and the use of physician-assessed phenotype variables in [49]. The model performance differed significantly; the AUC parameter describing model discrimination ranged from 0.66 for the model including only self-assessed risk factors without genotype information to 0.79 for the model including physician-assessed phenotype variables and genotype information. The main driver of the increase in the AUC was the use of physician-assessed nevus counts instead of self-assessed nevus information, while the use of genotype information only had a modest impact on the AUC.

Our large-scale investigation showed that even detailed instructions, including photographs explaining the definition of melanocytic nevi, i.e., which skin lesions should and should not be counted, failed to increase the level of agreement between self-assessed and examiner-assessed nevus counts, both without the assistance of epiluminescence microscopy, to an acceptable level of substantial or near-perfect agreement. The instructions and tools provided to the study population seem far more extensive than could typically be provided in a population setting or in a clinical setting. Together with the results of other studies, it seems questionable to use self-assessed information on phenotype variables such as the number of nevi when defining a high-risk subgroup of the population for targeted melanoma screening.

A promising development that might change this negative conclusion is the growing importance of artificial intelligence (AI) technology in dermatology [52,53]. A number of easy-to-use smartphone apps using new AI algorithms that can analyze skin images to identify and classify skin lesions accurately have been developed [54]. Proper validation of these AI algorithms is necessary—and currently on its way—before these tools can be used routinely. In the future, however, the use of this technology may allow self-assessment of skin lesions without dermatological expertise and may enhance precision in identifying high-risk individuals for targeted screening activities.

## 5. Conclusions

Our results show that even with comprehensive guidance, only a moderate level of agreement between self-assessed and examiner-assessed nevus counts, both without the assistance of epiluminescence microscopy, can be achieved. Self-assessed nevus information does not appear to be reliable enough to be used as a component in individual risk assessment for subsequent targeted screening programs.

## Figures and Tables

**Table 1:** Distribution of phenotype variables in the study sample (n = 4548).

Phenotype Variable	Absolute Number (n ^1^)	Proportion (%)
Fitzpatrick skin type		
Type I	234	5.18
Type II	1524	33.70
Type III	2352	52.01
Type IV	412	9.11
Freckling		
None	2632	58.02
Few	1546	34.08
Many	358	7.89
Hair color		
Red	61	1.34
Blonde	1214	26.75
Brown	2971	65.47
Black	292	6.44
Eye color		
Dark blue	496	10.94
Light blue/gray	1003	22.12
Green	847	18.68
Green/brown	1060	23.37
Light brown	610	13.45
Dark brown	519	11.44
Nevus score		
[0, 5]	1082	23.79
(5, 10]	940	20.67
(10, 15]	796	17.50
(15, 20]	561	12.34
(20, 30]	628	13.81
(30, 50]	415	9.12
>50	126	2.77

^1^ Absolute numbers of participants in the categories of the phenotypic variables do not add to the total sample size of 4548 due to missing information in some (8–26, depending on the variable) cases.

**Table 2:** Joint distribution of the five-level nevus score based on self-assessments (row variable) and examiner assessments (column variable) for all 4548 double-counting results. The main diagonal of the table comprises the absolute frequencies of agreement between self-assessed and examiner-assessed nevus score categories.

	Examiner Assessment					
		[0, 5]	(5, 10]	(10, 16]	(16, 26]	>26
Self-assessment	[0, 5]	695	152	37	12	0
	(5, 10]	251	380	166	51	12
	(10, 16]	87	218	285	165	21
	(16, 26]	38	135	292	368	115
	>26	11	55	132	311	559

**Table 3:** Agreement between self-assessed and examiner-assessed nevi counts in subgroups defined by sociodemographic and phenotype variables. Observed agreement (in %) and weighted kappa, both accompanied by 95% confidence intervals (CI), for the subgroups and result of the statistical evaluation of heterogeneity of subgroup estimates per variable.

Subgroup	Observed Agreement in % (95% CI)	Weighted Kappa (95% CI)	*p*-Value
Sex			0.08
Male	47.90 (45.52–50.28)	0.579 (0.554–0.604)	
Female	51.70 (49.87–53.54)	0.607 (0.588–0.626)	
Degree course			0.76
Clinical medicine	50.14 (48.62–51.66)	0.596 (0.580–0.611)	
Other	51.79 (46.84–56.73)	0.605 (0.554–0.655)	
Time			0.54
Summer term	50.25 (48.18–52.32)	0.601 (0.580–0.622)	
Winter term	50.33 (48.28–52.37)	0.592 (0.570–0.613)	
Fitzpatrick skin type			0.72
Type I	53.42 (47.03–59.81)	0.609 (0.542–0.677)	
Type II	49.87 (47.36–52.38)	0.585 (0.558–0.612)	
Type III	49.53 (47.51–51.55)	0.581 (0.560–0.603)	
Type IV	55.10 (50.29–59.90)	0.607 (0.556–0.658)	
Freckling			0.89
None	50.27 (48.36–52.18)	0.589 (0.569–0.609)	
Few	49.74 (47.25–52.23)	0.588 (0.561–0.614)	
Many	52.79 (47.62–57.97)	0.574 (0.514–0.633)	
Hair color			0.15
Red	52.46 (39.93–64.99)	0.561 (0.412–0.711)	
Blonde	48.11 (45.29–50.92)	0.565 (0.534–0.595)	
Brown	50.32 (48.52–52.12)	0.599 (0.581–0.618)	
Black	58.90 (53.26–64.55)	0.631 (0.571–0.691)	
Eye color			0.01
Dark blue	43.55 (39.18–47.91)	0.512 (0.462–0.561)	
Light blue/gray	48.75 (45.66–51.85)	0.590 (0.558–0.622)	
Green	51.71 (48.35–55.08)	0.606 (0.570–0.641)	
Green/brown	49.91 (46.90–52.92)	0.581 (0.548–0.613)	
Light brown	51.48 (47.51–55.44)	0.595 (0.553–0.638)	
Dark rown	56.84 (52.58–61.10)	0.642 (0.598–0.685)	

## Data Availability

The data presented in this study are available on request from the corresponding author.
